# Clinical characteristics and risk factors of Hepatitis E virus infection in Zhejiang Province: a multicenter case–control study

**DOI:** 10.3389/fpubh.2024.1417556

**Published:** 2024-07-05

**Authors:** Yijuan Chen, Jian Gao, Wanwan Sun, Weiping Zhu, Pingping Wang, Xiaobin Ren, Yumeng Wu, Jianli Zhang, Ziping Miao

**Affiliations:** ^1^Department of Communicable Disease Control and Prevention, Zhejiang Provincial Center for Disease Control and Prevention, Hangzhou, China; ^2^Department of Communicable Disease Control and Prevention, Qujiang District Center for Disease Control and Prevention, Quzhou, China; ^3^Pujiang County Center for Disease Control and Prevention, Jinhua, China; ^4^Department of Communicable Disease Control and Prevention, Hangzhou Center for Disease Control and Prevention, Hangzhou, China; ^5^Department of Communicable Disease Control and Prevention, Jiashan County for Disease Control and Prevention, Jiaxing, China; ^6^Department of Communicable Disease Control and Prevention, Wenling City Center for Disease Control and Prevention, Taizhou, China

**Keywords:** hepatitis E, risk factors, clinical characteristics, prevention and control, case-control study

## Abstract

**Introduction:**

Hepatitis E (HE), caused by the Hepatitis E virus (HEV), is a significant cause of acute viral hepatitis globally and a major public health concern, particularly in specific high-prevalence areas in China, which have diverse transmission routes and regional differences. Identifying the primary risk factors for HE transmission is essential to develop targeted interventions for vulnerable populations.

**Methods:**

This study employed a 1:1 matched case–control methodology, using a standardized questionnaire complemented by medical records for data validation.

**Results:**

Among the 442 HE cases and 428 healthy controls, the case group had a higher prevalence of fatigue (46.21%) and loss of appetite (43.84%) compared to the control group. Furthermore, liver function indicators were significantly higher in the case group, with an average alanine aminotransferase (ALT) level of 621.94 U/L and aspartate aminotransferase (AST) level of 411.53 U/L. Severe HE patients were predominantly male, with significantly increased ALT and AST levels reaching 1443.81 U/L and 862.31 U/L respectively, along with a higher incidence of fatigue (90%) and loss of appetite (75%). Multifactorial analysis indicated that frequent dining out (OR = 2.553, 95%CI:1.686–3.868), poor hygiene conditions (OR = 3.889, 95%CI:1.399–10.807), and comorbid chronic illnesses (OR = 2.275, 95%CI:1.616–3.202) were risk factors for HE infection; conversely, good hygiene practices were protective factors against HE infection (OR = 0.698, 95%CI:0.521–0.934).

**Conclusion:**

In conclusion, HE infection in Zhejiang Province is closely associated with dietary habits and environmental hygiene, and individuals with chronic diseases or co-infections are at increased risk. This highlights the need for targeted health education to reduce the incidence of HE among these populations.

## Introduction

Hepatitis E (HE), a significant cause of acute viral hepatitis, is primarily caused by the hepatitis E virus (HEV), a quasi-enveloped virus with a single-stranded positive RNA genome belonging to the hepatitis virus family. HE is characterized by inflammatory necrosis of liver parenchymal cells, presenting symptoms similar to hepatitis A, but tends to lead to jaundice and more severe conditions, with a longer illness duration and a higher mortality rate of around 3% (1). The disease is particularly threatening to vulnerable populations, such as pregnant women, the older adults, and individuals with chronic hepatitis. The mortality rate for infected pregnant women can reach up to 30% (2). Individuals with chronic liver disease who acquire HE have a heightened risk of developing liver failure, with mortality rates reaching up to 70%. Additionally, immunosuppressed individuals, such as organ transplant recipients, may develop chronic infections following HEV infection (3, 4).

HEV is transmitted through various routes, which primarily include fecal-oral, bloodborne, vertical transmission, and close contact. Fecal-oral transmission, the most common route, includes waterborne and foodborne mechanisms. Waterborne transmission occurs through fecal and urine contamination of water sources, while foodborne transmission happens via the ingestion of food or raw animal products containing HEV (5). The global epidemiology of HE can be divided into two distinct modes: waterborne (HEV genotypes 1 and 2) and zoonotic (HEV genotypes 3 and 4) (6). In high-incidence regions, transmission often occurs through contaminated water supplies, leading to inter-individual spread. Conversely, in high-incidence regions, HE primarily arise from zoonotic transmission, which occurs due to close contact with infected animals or through the consumption of contaminated food products, most commonly raw or undercooked meat (7). In China, HEV4 has recently surpassed HEV1 as the dominant genotype, likely due to improved sanitation and hygiene measures (8).

Since the discovery of HE, outbreaks have occurred globally, with the largest epidemic affecting 119,280 people and resulting in 707 deaths in southern Xinjiang, China, from 1986 to 1988 (9). While HE outbreaks are common in developing countries with inadequate sanitation, industrialized nations have recently experienced an increasing number of locally acquired sporadic cases. The Global Burden of Disease study estimates that one-third of the world’s population is at risk of HEV infection, which is responsible for 70,000 deaths and 3,000 stillbirths annually (10). China is a highly endemic area for HE, with the reported incidence rate increasing from 1.27 per 100,000 in 2004 to 2.11 per 100,000 in 2023 (11). A meta-analysis of 208 studies involving 1,785,569 subjects revealed that the seroprevalence of anti-HEV IgG in China was 23.17%, with Zhejiang Province having a seropositivity rate of 37.24% (11). From 2004 to 2022, Zhejiang Province reported 37,494 cases of HE, ranking among the top three in China. The proportion of reported HE cases among viral hepatitis cases increased annually from 2.88 to 13.78%, while the proportion among acute viral hepatitis cases rose from 12.77 to 86.55% (unpublished data).

Overall, the prevention and control of HE are crucial public health issues in Zhejiang Province, China. However, the clinical characteristics and transmission risk factors of HE infections in this region are poorly understood. This study aims to investigate the clinical features, transmission patterns, and risk factors of HEV infection in Zhejiang Province to identify high-risk groups and provide evidence-based insights for developing targeted prevention and control strategies, ultimately contributing to the improvement of public health in the region.

## Materials and methods

### Design of the study and research location

**Figure 1:**
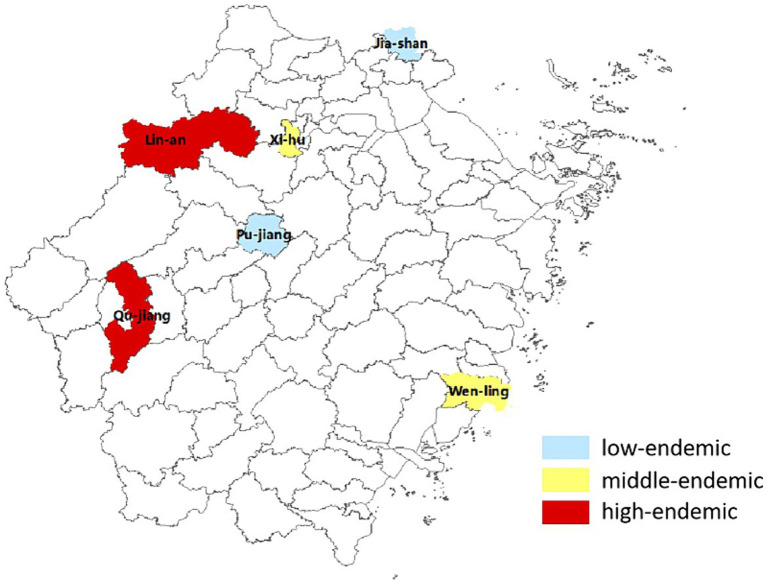
Spatial distribution of HE incidences in Zhejiang province.

Located in the eastern coastal area of China, Zhejiang Province covers an area of approximately 105,000 km^2^. This is comparable in size to Portugal and Hungary. Our multicenter case–control study began in July 2015 across six diverse areas within Zhejiang - West Lake in Hangzhou, Lin’an, Jiashan, Qujiang, Wenling, and Pujiang - which were selected based on their differences in geographical, economic, and historical incidence rates (Figure 1).

### Definition and identification of cases

**Case selection** Laboratory-confirmed cases of HE reported between January 2014 and December 2022 were included from each region, with cases from January 2014 to July 2015 collected retrospectively. Medical institutions in the study area diagnosed and clinical classified individuals infected with HEV, following the ‘Guidelines for the Diagnosis and Treatment of Hepatitis E (2009 Edition)’ (12).

**Control selection** Concurrently, for each case, we selected one control residing in close proximity to the case and not exhibiting symptoms of hepatitis. The controls were required to meet the following criteria: no prior history of hepatitis A or E, negative serum HEV IgM antibody results, being of the same gender as the corresponding cases, and an age difference within 5 years.

### Cases definition

**Severe Cases** Patients present with symptoms such as severe fatigue, anorexia, vomiting, and significant gastrointestinal symptoms, including abdominal distension. Patients experience progressive worsening of jaundice, with serum total bilirubin levels exceeding 171 μmol/L (10 mg/dL) and prothrombin activity≤40%. Potential complications include hepatic encephalopathy, ascites, hepatorenal syndrome, and upper gastrointestinal bleeding.

**Mild Cases** Patients meet the diagnostic criteria outlined in the ‘Guidelines for the Diagnosis and Treatment of Hepatitis E (2009 Edition)’ but do not reach the severity standards defined for severe cases.

### Survey method

Epidemiological investigations were conducted using a “Survey on HE Cases in Zhejiang Province” questionnaire for both cases and controls. The questionnaire collected information on demographics, medical history, risk behaviors, clinical symptoms, disease progression, consultations, and lab results, including liver function. The survey covered the following aspects: (1) General information such as gender, age, occupation, ethnicity, family, and vaccination status; (2) Risk factors such as water sources and dietary habits (eating out frequency, food types, vendor choice). It also included exposure history (contact with hepatitis patients, animals, travel encounters), environmental hygiene (waste and excreta disposal, livestock pen presence, fly exposure, animal health), and personal lifestyle (handwashing, shared drinking habits, unboiled water and alcohol consumption, home cleaning, window airing); (3) Detailed case investigations, including retrospective collection of laboratory data.

Serum samples were collected from both cases and controls. Hepatitis antibodies were detected using enzyme-linked immunosorbent assay (ELISA), while liver function tests, including alanine aminotransferase (ALT), aspartate aminotransferase (AST), and total bilirubin, were measured using standard blood biochemical assays. The same methods were applied to both groups to ensure consistency and comparability of the results.

### Statistical analysis

The data were entered using Epidata 3.0 and subsequently transferred to Excel for the purpose of creating a database. Statistical analyses, including chi-square tests, t-tests, ANOVA, and multivariable conditional logistic regression, were conducted using SPSS 26.0 software with a significance level set at *p* < 0.05. Ordered categorical variables initially treated as continuous were included in the multivariable analysis as dummy variables if found to be significant. Variables with a significance level below *p* < 0.2 were selected for inclusion in the multivariate analysis utilizing the forward LR method.

### Ethical statement

This study was conducted in response to a public health emergency and received approval from the ethics committee of Zhejiang Provincial Centre for Disease Control and Prevention, with the ethics number of 2,016,020. Informed consent was obtained from all participants or their legal guardians. The study was conducted in accordance with the Declaration of Helsinki.

## Results

### Descriptive epidemiology

The study comprised a total of 870 participants, with 442 cases and 428 controls. The majority (47.29%) were from the West Lake District in Hangzhou. Cases had a mean age of 52.23 ± 15.27 years, while controls had a mean age of 50.53 ± 15.03 years. The male-to-female ratio among cases was 1.82:1. The predominant occupations were farmers (24.21%), industrial workers in factories (14.93%), homemakers/unemployed individuals (11.99%), cadres/officers (11.54%), and business service staff (11). Age, gender, and education levels were similar between cases and controls (Table 1).

**Table 1:** Demographic characteristics of participants in the case–control study.

	Case (*n* = 442)	Control (*n* = 428)	Statistics	*p* value
Regions			0.298	0.998
West Lake District	209	209		
Lin’an	51	49		
Jiashan	41	37		
Qujiang	83	77		
Wenling	39	37		
Pujiang	19	19		
Age	52.23 ± 15.27	50.53 ± 15.03	1.652	0.968
Gender			3.382	0.066
Male	285(64.48%)	250 (58.41%)		
Female	157 (35.52%)	178 (41.59%)		
Educational level			4.519	0.104
Elementary level or below	144 (32.58%)	112 (26.17%)		
Junior or senior high school	209 (47.29%)	227 (53.04%)		
University or higher	89 (20.14%)	89 (20.79%)		

### Clinical manifestations and clinical laboratory results

#### Clinical manifestations

**Figure 2:**
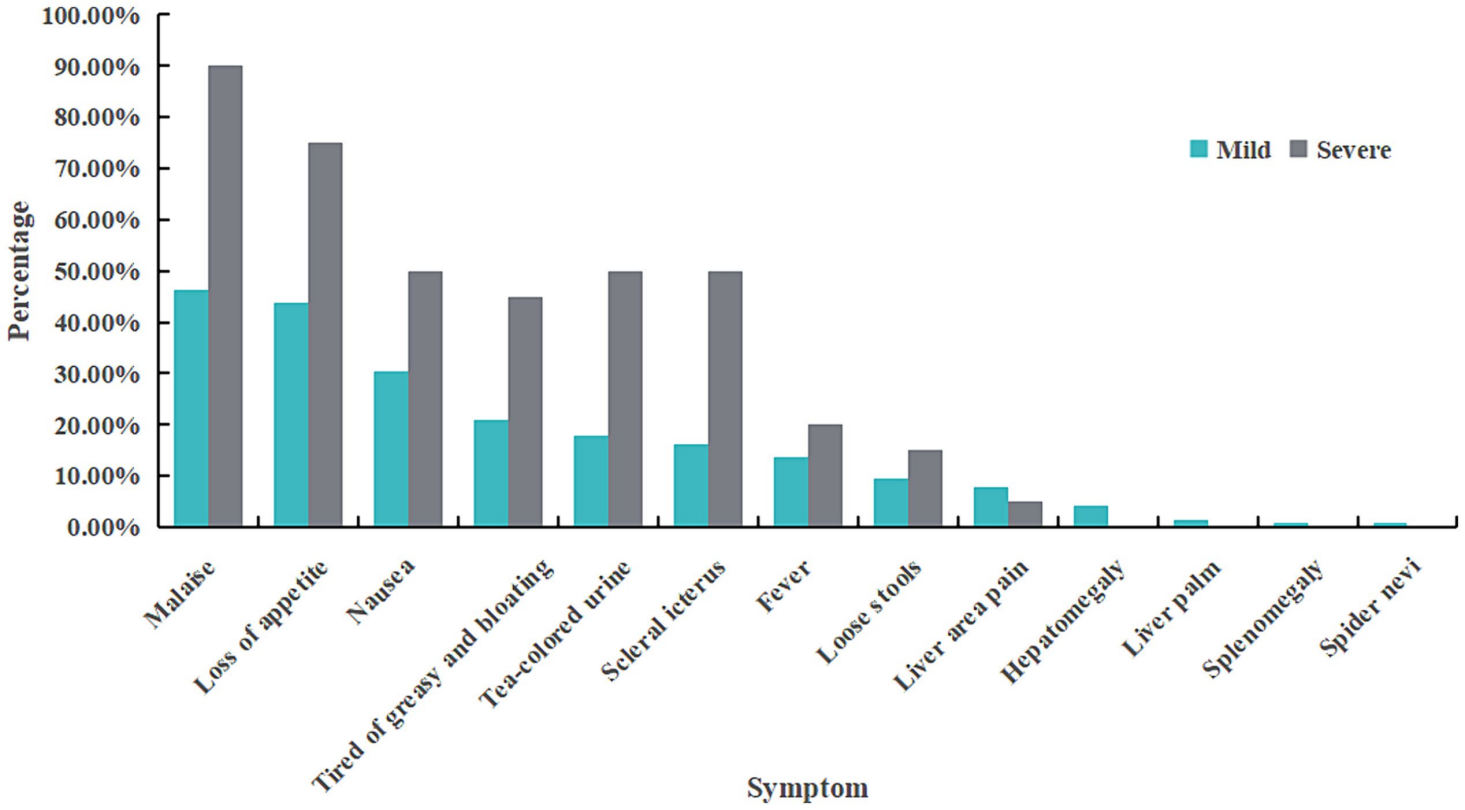
Symptom distribution of HE cases with different severity.

Among 442 cases, 422 were classified as mild while the remaining 20 were categorized as severe. Severe cases were more prevalence among males (*p* = 0.015). However, age did not significantly differ between severe and mild cases (*p* = 0.681). The most common symptoms reported across all cases were fatigue (48.19%), appetite loss (45.25%), nausea (31.22%), aversion to oily food and bloating (21.95%), dark urine (19.23%), jaundice (17.65%), fever with some cases reaching up to 39°C(14.03%), diarrhea (9.73%), liver pain (7.69%), liver enlargement (4.07%), liver palm sign, spleen enlargement, and spider angiomas were also observed in this study (Table 2; Figure 2).

**Table 2:** Clinical and laboratory characteristics of participants with different severity.

	Mild cases (*n* = 422)	Severe cases (*n* = 20)	All cases (*N* = 442)	Controls (*N* = 428)
Male Proportion (%)	63.27%	90.00%	64.48%	/
Age(mean (range))	52.15 (19–94)	53.60 (25–80)	52.21 (19–94)	/
Symptom (n(%))				
Malaise	195 (46.21%)	18 (90.00%)	213 (48.19%)	/
Loss of appetite	185 (43.84%)	15 (75.00%)	200 (45.25%)	/
Nausea	128 (30.33%)	10 (50.00%)	138 (31.22%)	/
Tired of greasy and bloating	88 (20.85%)	9 (45.00%)	97 (21.95%)	/
Tea-colored urine	75 (17.77%)	10 (50.00%)	85 (19.23%)	/
Scleral icterus	68 (16.11%)	10 (50.00%)	78 (17.64%)	/
Fever	58 (13.74%)	4 (20.00%)	62 (14.03%)	/
Loose stools	40 (9.48%)	3 (15.00%)	43 (9.73%)	/
Liver area pain	33 (7.82%)	1 (5.00%)	34 (7.69%)	/
Hepatomegaly	18 (4.27%)	0 (0.00%)	18 (4.07%)	/
Liver palm	6 (1.42%)	0 (0.00%)	6 (1.36%)	/
Splenomegaly	4 (0.95%)	0 (0.00%)	4 (0.90%)	/
Spider nevi	3 (0.71%)	0 (0.00%)	3 (0.68%)	/
Laboratory test results (n(%))				
ALT (U/L)^*^	552.60 (10.5–9,613),256	1509.5 (85–4,260),20	621.94 (10.5–9,613),276	23.72 (6–51),86
AST (U/L)^*^	333.14 (12–5,091),112	862.31 (40–2,787),16	411.53 (12–5,091),128	20.91 (0–42),55
Total bilirubin (μmol/L)^*^	58.75 (5.9–901),115	246.22 (171–602),20	86.52 (5.9–901),135	15.02 (4.2–142),54
HBsAg	7 (1.66%)	2 (0.00%)	9 (2.04%)	0 (0.00%)
Anti-HBs	9 (2.13%)	1 (5.00%)	10 (2.26%)	2 (0.47%)
HBeAg	1 (0.24%)	1 (5.00%)	2 (0.45%)	0 (0.00%)
Anti-HBe	5 (1.18%)	0 (0.00%)	5 (1.13%)	0 (0.00%)
Anti-HBc IgM	10(%)	0 (0.00%)	10 (2.26%)	0 (0.00%)

The symbol “*” indicates that the data in the corresponding cell are presented as mean (range), and the total number of participants who underwent the relevant test.

Among the cases, 147 (33.26%) presented with chronic conditions, predominantly hypertension and diabetes. Among these individuals, 21 individuals had liver diseases, which included 11 with hepatitis B, 4 with fatty liver disease, 2 with alcoholic liver disease, 2 with cirrhosis, 1 with autoimmune hepatitis, and 1 with an unspecified liver condition. Among the controls, chronic diseases were observed in 85 individuals (19.86%), primarily hypertension and diabetes. Two controls had liver-related comorbidities: one had schistosomiasis while another had an unspecified liver disorder.

#### Clinical laboratory results

Blood sample analysis revealed that the average level of ALT in the case group was 621.94 U/L, with 96.38 and 80.07% of cases exceeding 40 U/L and 80 U/L, respectively; the average AST level was 411.53 U/L, with 83.33% above 40 U/L and 58.33% above 80 U/L; additionally, the mean total bilirubin concentration was measured at 86.52 μmol/L, with an alarming rate of 72.59% exceeding 17.1 μmol/L. Critically ill cases had significantly higher levels of ALT(1509.5 U/L), AST(862.31 U/L), and bilirubin(246.22 μmol/L). In contrast, the control group had much lower average levels of ALT (23.72 U/L), AST(20.91 U/L), and bilirubin (15.02 μmol/L; Table 2).

In the case group, 9 individuals were HBsAg(+), 10 were anti-HBs(+), 2 were HBeAg(+), 5 were anti-HBe(+), and 10 were anti-HBc-IgM(+). These findings suggest that a total of 10 subjects had co-infection with HE and hepatitis B (Table 2).

### Analysis of risk factors

#### Single-factor analysis of HE risk factors

Preliminary single-factor analysis revealed statistically significant differences between the two groups in several aspect. These include the presence of chronic underlying diseases, frequency of dining out (occasionally or often compared to not at all), consumption from mobile food stalls within the past 6 months, patronage of fast food restaurants and rural eateries, presence of livestock pens at home or neighbor’s residence, presence of flies at home prior to illness, regular sanitation practices, and occasional sharing of water cups and tableware compared to abstaining from such practices (Table 3).

**Table 3:** Univariate analysis of risk factors for HE^#^.

Exposure factor	Case		Control		OR	95%CI	*p* value
	+	−	+	−			
Having chronic underlying illness	147	295	85	343	2.011	1.476–2.739	0.000
Eating out in the past 6 months (Using “no” as a reference)	198		196				0.008
Rarely	84		84		1.237	0.857–1.787	0.255
Occasionally	93		80		1.439	0.999–2.071	0.050
Often	100		66		1.875	1.290–2.726	0.001
Recently dined at a mobile food stall within the past 6 months	40	378	22	391	1.881	1.097–3.224	0.022
Recently dined at a fast food restaurant within the past 6 months	120	298	88	325	1.487	1.083–2.042	0.014
Recently dined at a farmhouse restaurant in the past 6 months	30	388	15	398	2.052	1.087–3.873	0.027
Recently dined at a sidewalk snack booth within the past 6 months	35	383	26	387	1.360	0.803–2.303	0.252
Recently dined at other restaurants in the past 6 months*	143	269	130	274	1.120	0.838–1.499	0.444
Regular consumption of cured meats	175	260	173	255	0.993	0.860–1.146	0.921
Regular consumption of delicatessen and cold dishes	94	341	84	343	1.061	0.887–1.269	0.515
Regular consumption of seafood	256	179	252	172	1.016	0.904–1.143	0.789
Regular consumption of freshwater products	295	140	297	127	0.985	0.879–1.104	0.794
Regular consumption of barbecue food	89	349	95	330	0.972	0.833–1.135	0.722
Regular consumption of pork or pork offal	274	161	253	171	1.054	0.942–1.180	0.360
Regular consumption of pig liver	145	115	121	120	1.250	0.880–1.777	0.213
Recently consumed uncooked pig liver in the past 6 months	10	425	5	409	1.925	0.652–5.679	0.236
The presence of a livestock enclosure at our/our neighbor’s residence	74	358	49	375	1.582	1.072–2.334	0.021
Prior to the onset of the illness, there was a presence of flies within the premises	220	215	155	236	1.589	1.240–2.036	0.000
Regularly clean the house	201	232	236	181	0.664	0.507–0.871	0.003
Practicing hand hygiene by washing hands before meals and after using the washroom	19	415	12	412	0.636	0.305–1.327	0.228
Sharing water cups and utensils (Using “no” as a reference)	328		347				0.073
Occasionally	68		47		1.531	1.025–2.286	0.038
Often	39		32		1.289	0.789–2.107	0.311
History of hepatitis exposure	13	429	7	421	1.823	0.720–4.613	0.205
Travel History	16	426	9	419	1.749	0.764–4.001	0.186
Drinking untreated or unfiltered water	20	413	24	401	0.869	0.558–1.353	0.535
Drinking alcohol	151	291	125	303	1.108	0.972–1.263	0.126
Smoking	135	279	122	283	1.106	0.932–1.313	0.249

^#^Due to the extensive analysis of multiple variables, only primarily primary variables are presented. No statistically significant results were observed for the unreported subclass variables. *Including large and medium-sized restaurants, themed restaurants, and Western restaurants.

#### Multi-factor analysis of HE risk factors

The results of a multifactorial analysis indicate that the primary risk factors for HE infection are frequent dining out, presence of flies at home prior to illness, regular household cleaning practices, and the presence of a chronic underlying disease. Compared to abstaining from dining out, the risks associated with infrequent dining out, occasional dining out, and frequent dining out are 1.701-fold, 2.128-fold, and 2.553-fold higher, respectively, demonstrating a clear dose–response relationship. The risks of occasional and frequent presence of flies at home are 1.524-fold and 3.889-fold higher, compared to the absence of flies at home, respectively, also demonstrating a clear dose–response relationship. Regular household cleaning reduces a 0.698-fold lower risk compared to irregular cleaning practices. Individuals with chronic underlying diseases have a significantly elevated risk (2.275 times) compared to those without such conditions (Table 4).

**Table 4:** Multi-factor analysis of risk factors for HE.

	*β*	S_E_	Wald	*p* value	OR	95% *CI*	
Eating out in the past 6 months (Using “no” as a reference)			24.957	0.000			
Rarely	0.531	0.203	6.833	0.009	1.701	1.142	2.533
Occasionally	0.755	0.207	13.278	0.000	2.128	1.417	3.193
Often	0.937	0.212	19.572	0.000	2.553	1.686	3.868
Prior to the onset of the illness, there was a presence of flies within the premises (Using “no” as a reference)			12.567	0.002			
Occasionally	0.421	0.153	7.546	0.006	1.524	1.128	2.058
Often	1.358	0.521	6.783	0.009	3.889	1.399	10.807
Regularly clean the house (Using “no” as a reference)	−0.36	0.149	5.838	0.016	0.698	0.521	0.934
Having chronic underlying illness (Using “no” as a reference)	0.822	0.174	22.192	0.000	2.275	1.616	3.202

## Discussion

HEV, as the main cause of acute viral hepatitis (13), infects about a third of people globally (14). The prevalence of HEV infection varies depending on viral genotypes (15). In China, genotype 4 has emerged as the dominant strain since 2000, supplanting genotype 1 (16). Notably, Zhejiang Province exhibits a high incidence of HEV infections, reporting high HEV incidence, with 45.54% of the rural population showing HEV-IgG antibodies, well above the national 23.17% average (11), and even higher (63.60%) in high-risk jobs (17). Therefore, understanding the clinical characteristics and transmission of HEV in Zhejiang is vital for targeted disease prevention and control.

The study findings suggest that HE infection is association with frequent dining out, suboptimal environmental hygiene practices, and unhealthy behavioral habits. Furthermore, a detailed analysis of different dining establishments reveals a higher risk of HE infection among individuals who consume food from street stalls, fast-food outlets, and food stands. Conversely, the risk is relatively lower for those who dine in medium to large restaurants and themed eateries. These results suggest potential disparities in food safety regulations and environmental hygiene standards across various types of restaurants that may contribute to the varying risks associated with HE infection. Additionally, numerous studies have demonstrated that pigs serve as significant reservoirs for HEV, and the consumption of undercooked pig liver poses a high-risk factor for HEV infection (18, 19). In this study, the proportion of cases who consumed undercooked pig liver (10/442) was 1.925 times higher than that in the control group (5/428), although this difference did not reach statistical significance. This difference may be due to the relatively limited number of participants who reported consuming undercooked pig liver. In Zhejiang Province, HE is mainly zoonotic (HEV-3, HEV-4), with genotype 4 being dominant post-2000. Our study on HE prevalence examined water and contact transmission. We found no significant link, implying lower risks from water and interpersonal spread, and higher risks from food. Control efforts should thus target food hygiene, including sourcing, transport, processing, and sales, along with pest management and personal cleanliness. Enhanced health education and a focus on prevention are key to managing the spread of HE.

HEV infection can present both clinically and subclinically (20). The study revealed a higher prevalence of clinical HEV cases in middle-aged/older adults, with males exhibiting a rate 1.82 times higher than that of females. Approximately one-third (33.48%) of individuals had underlying chronic conditions, which may increase their risk of HEV infection. Common symptoms, such as fatigue and anorexia, can lead to frequent instances of underdiagnosis or misdiagnosis. Cases demonstrated significantly elevated levels of ALT, AST, and bilirubin compared to controls. Further analysis revealed that 5.88% of cases (26/442) exhibited liver diseases. Additionally, 2.26% (10/442) of cases also presented with hepatitis B infection, in contrast to controls where only 0.47% (2/428) had liver diseases and none were infected with hepatitis B. These findings suggest that co-infections may enhance the risk of HEV illness, which is consistent with the findings of previous studies (21). Severe HEV cases demonstrated a male-to-female ratio of 9:1. While HEV generally exhibits normal laboratory results, HEV poses a higher risk for severe disease compared to Hepatitis A, particularly among older populations or individuals with underlying liver conditions or compromised immunity. Notably, our study identified severe HEV cases in 4.52% of participants who often presented with chronic diseases or co-infections, emphasizing the necessity for targeted monitoring of these vulnerable groups.

The findings of this study have certain limitations that may have influenced the results: Firstly, the absence of a double-blind design could introduce investigator bias. Secondly, due to the prolonged incubation period and disease progression of HE, it is challenging to gather pertinent information at the onset of illness. As a result, the survey primarily focused on participants’ dietary and drinking preferences rather than their actual living conditions prior to the onset of illness.

Our study explores the clinical traits and risks of HE in Zhejiang. The findings can inform improved prevention and public health strategies. The results link HE infection to diet and sanitation, emphasizing the importance of these factors. Individuals with chronic diseases or co-infections are at a particularly high risk, highlighting the need for focused health education to mitigate these risks.

## Data availability statement

The raw data supporting the conclusions of this article will be made available by the authors, without undue reservation.

## Ethics statement

The studies involving humans were approved by the ethics committee of Zhejiang Provincial Centre for Disease Control and Prevention. The studies were conducted in accordance with the local legislation and institutional requirements. Written informed consent for participation in this study was provided by the participants’ legal guardians/next of kin.

## Author contributions

YC: Investigation, Methodology, Software, Supervision, Writing – original draft, Writing – review & editing. JG: Investigation, Project administration, Supervision, Writing – review & editing. WS: Data curation, Investigation, Methodology, Software, Writing – review & editing. WZ: Project administration, Supervision, Writing – review & editing. PW: Project administration, Supervision, Writing – review & editing. XR: Project administration, Supervision, Writing – review & editing. YW: Project administration, Supervision, Writing – review & editing. JZ: Project administration, Supervision, Writing – review & editing. ZM: Conceptualization, Project administration, Resources, Supervision, Writing – original draft.
