# Supplementary material for: Clinical characteristics and risk factors of Hepatitis E virus infection in Zhejiang Province: a multicenter case–control study
Source: Front Public Health. 2024 Jul 5;12:1417556. doi: 10.3389/fpubh.2024.1417556 (PMC11257836; doi:10.3389/fpubh.2024.1417556)
Supplement: Supplementary file 1 [file Data_Sheet_1.docx]

**Apendix 1.**  **Hepatitis E Case Investigation Form, Zhejiang Province**

Grouping of Objects Case口 Control口 (Corresponding case name________)

Case Number □□□□□□□□

General Information

1. Name: ________; if the individual is under 14 years old, the parent's name is ________

2. ID Number: __________________ (15 or 18 digits)

3. Gender: ①male ②female

4. Date of Birth: ____year_____month_____day

if the date of birth is unknown, then enter the actual age:_____ Age unit: ①year ②month ③day)

5. Employer: _________________________________________________________

6. Contact Number: ___________

7. Current Address: ①This county district ②Other county/district of the city ③Other cities in the province ④Other provinces ⑤Hong Kong, Macao, and Taiwan ⑥Foreign nationality

8. Current Address (Please fill in the details): ______ Province ________ City__________ County (District) __________ Township (Town, Street)____________ Village (Administrative Village)

9. Is it the usual residence ①yes (more than or equal to 6 months) ②no

10. Residential Area: ①urban area ②urban-rural fringe ③suburban area ④rural area

11. Occupation:

①Preschool children ②Scattered children ③Students (primary, middle, and high school) ④Teachers

⑤Caregivers and nannies ⑥Food and beverage industry ⑦Commercial services ⑧Medical personnel

⑨Workers ⑩Migrant workers ⑪Farmers ⑫Herders ⑬Fishermen (boat) ⑭Cadres and staff

⑮Retired personnel ⑯Housework and unemployed ⑰Other occupations ⑱Slaughtering industry (specify)____________ ⑲Breeding industry (specify)____________ ⑳Not specified

Years of experience in the profession: ____________

Is there a need to come into contact with pigs (or their internal organs)? ①Yes ②No

12. Education level: ①Illiterate ②Preschool children ③Primary school ④Junior high school ⑤High school or technical secondary school ⑥University and above ⑦Not specified

13. Ethnicity: ①the Han nationality ②Other

14. Do you smoke? ①Often ②Occasionally ③No (If it is a control, skip to 19)

15. Case classification: ①suspected case ②clinically diagnosed case ③laboratory diagnosed case

④pathogen carrier

16. Date of onset: ____year_____month_____day (fill in the date of diagnosis for pathogen carriers)

17. Initial diagnosis date:____year_____month_____day

Initial Diagnosis Institution: ____________________

1. Diagnosis date:____year_____month_____day

Diagnosis Institution: ____________________

19. Have you had any illness in the past six months? ①cold ②diarrhoea ③other

20. History of hepatitis A or B vaccination: ①yes ②no ③unknown

If yes, vaccination dates: First dose ______year______ month Second dose______year______ month

21. Total family members: ____ (refers to the number of people who have had meals together for most of the time in the past 3 months)

Average annual family income: ①Below 10,000 yuan ②10,000-30,000 yuan ③30,000-50,000 yuan ④50,000-100,000 yuan ⑤Above 100,000 yuan

Clinical Manifestations

1. Decreased appetite: ①Yes ②No ③Unknown

2. Nausea: ①Yes ②No ③Unknown

3. Aversion to oily foods and abdominal distension: ①Yes ②No ③Unknown

4. Loose stools: ①Yes ②No ③Unknown

5. Fatigue: ①Yes ②No ③Unknown

6. Tea-colored urine: ①Yes ②No ③Unknown

7. Pain in the liver area: ①Yes ②No ③Unknown

8. Fever: ①Yes ②No ③Unknown If yes, the highest body temperature: _______ ℃

9. Hepatomegaly (enlarged liver): ①Yes ②No ③Unknown

10. Splenomegaly (enlarged spleen): ①Yes ②No ③Unknown

11. Jaundice of the sclera: ①Yes ②No ③Unknown

12. Spider nevi: ①Yes ②No ③Unknown

13. Palmar erythema: ①Yes ②No ③Unknown

14. Other symptoms:_________________________

15. Suffering from chronic diseases: ①Yes ②No

If yes, medical institution diagnosed disease name: _____________________________

16. Course and outcome: ___________________________________

Laboratory Test Results (Sample Collection Date:____year_____month_____day )

1 ALT________ U/L

2 AST________ U/L

3 Total Bilirubin_____________umol/L

4 Anti-HAV-IgM ①＋ ②－

5 HBsAg ①＋ ②－

6 Anti-HBs ①＋ ②－

7 HBeAg ①＋ ②－

8 Anti-HBe ①＋ ②－

9 Anti-HBc-IgM ①＋ ②－

10 Anti-HCV-IgM ①＋ ②－

11 Anti-HDV-IgM ①＋ ②－

12 Anti-HEV-IgM ①＋ ②－

13 Anti-HEV-IgG ①＋ ②－

14 Others:________________________________________________________________________

Investigation of Relevant Factors

(I) Dietary History (Comparison and inquiry about the corresponding time with the case)

1. Do you often dine out? ①Yes (1-2 times/week) ②Occasionally (1-2 times/month) ③Rarely (<1 time/month) ④No

1.1 If dining out, please specify the types of dining places (multiple choices allowed):

①Mobile food stalls ②Street food stalls ③Fast food restaurants ④Large-scale restaurants

⑤Medium-sized restaurants ⑥Themed restaurants ⑦Western restaurants ⑧Farmhouse restaurants

1. Do you often consume cured meats?

①Yes (1-2 times/week) ②Occasionally (1-2 times/month) ③Rarely (<1 time/month) ④No

2.1 If yes, please specify the specific types:____________________________________________;

Purchase locations (multiple choices allowed):

①Mobile food stalls ②Farmers' market stalls ③Branded cured meat stores

④Other (please specify) ____________________

3. Do you often consume ready-to-eat cold dishes (such as cold noodles, cold jelly, and other foods that do not require heating)?  ①Yes (1-2 times/week) ②Occasionally (1-2 times/month) ③Rarely (<1 time/month) ④No

3.1 If yes, please specify the specific types: __________________________________________;

Purchase locations (multiple choices allowed):

①Mobile food stalls ②Farmers' market stalls ③Ready-to-eat food stores

④Other (please specify) ________________________

4. Do you often consume seafood? ①Yes (1-2 times/week) ②Occasionally (1-2 times/month) ③Rarely (<1 time/month) ④No

4.1 If yes, please specify the specific types: ①Shellfish ②Shrimp ③Snails ④Crab ⑤Other

5. Do you often consume freshwater products? ①Yes (1-2 times/week) ②Occasionally (1-2 times/month) ③Rarely (<1 time/month) ④No

5.1 If yes, please specify the specific types: ①Shellfish ②Crayfish ③Snails ④Crab ⑤Other

6. Do you often consume barbecue foods? ①Yes (1-2 times/week) ②Occasionally (1-2 times/month) ③Rarely (<1 time/month) ④No

6.1 If yes, please specify the specific types: ①Beef skewers ②Lamb skewers ③Pork skewers

④Chicken skewers ⑤Other

7. Do you often consume pork or pork offal? ①Yes (1-2 times/week) ②Occasionally (1-2 times/month) ③Rarely (<1 time/month) ④No

7.1 If yes, please specify the specific type: ①Pork heart ②Pork liver ③Pork intestines

④Pork stomach ⑤Pig tongue ⑥Other

7.2 Multiple choices for consumption method: ①Steamed ②Braised ③Stewed ④Fried ⑤Boiled

⑥Deep-fried ⑦Braised in soy sauce ⑧Simmered ⑨Stir-fried ⑩Shabu-shabu (⑪Soup-making ⑫Roasted

8. Before the illness, did you consume undercooked pork liver (tender pork liver)? ①Yes ②No

8.1 If yes, approximately how long ago? ①Within 1 week ②Within 2 weeks ③Within 3 weeks ④Within 1 month ⑤Within 2 months ⑥More than 2 months

9. Consumption of raw fruits and vegetables: ①Watermelon ②Cucumber ③Grapes ④Tomato ⑤Apple ⑥Pear ⑦None ⑧Other

9.1 Washing method: ①Rinsing with water ②Wiping with hands or cloth ③Peeling before consumption ④Direct consumption ⑤Other

10. What do you suspect as the risk factors that led to the illness?_________________

(II)Drinking Water and Environmental Conditions

1. Drinking water source: ①Piped tap water ②Other tap water ③Spring water ④Deep well water ⑤Shallow well water ⑥River water ⑦Pond water ⑧Other_______________

2. Washing water: ①Piped tap water ②Other tap water ③Spring water ④Deep well water ⑤Shallow well water ⑥River water ⑦Pond water ⑧Other_______________

3. Household waste disposal: ①Sent to landfill ②Thrown outdoors ③Buried

④Other ________________________

4. Faecal waste disposal: ①Sanitary toilet ②Municipal sewage system ③Used as fertilizer for crops ④Direct discharge ⑤Other_______________________

5. Do you or your neighbours have livestock pens? ①Yes ②No

If yes, you can select multiple: ①Pigs ②Dogs ③Cats ④Rats ⑤Chickens ⑥Ducks

⑦Other ___________________________________;

6. Were there flies in the house before the illness? ①Frequently ②Occasionally ③None

7. Did the animals show any signs of illness or unusual deaths before the illness? ①Yes ②No

(III) History of Contact (Contact persons for comparison are not counted as corresponding cases)

1. Before the onset of the illness (or within the past year), have you had any contact with other hepatitis patients? ①No ②Yes ③Unclear

If yes, type of hepatitis: ①Hepatitis A ②Hepatitis B ③Hepatitis C ④Hepatitis D

⑤Hepatitis E ⑥Hepatitis (unclassified) ⑦Other: ______________________________

Relationship with the patient: ______________________

Method of contact: ①Eating together ②Living together ③Sharing daily life

④Working together ⑤Studying together ⑥Playing together ⑦Caregiving

2. Among your colleagues at work, have any had hepatitis A or E? ①No ②Yes ③Unclear

3. Close contacts registration:

| Name | Gender | Age | Relationship | Company or address | Affected by the illness | Date of onset | Laboratory test |
| --- | --- | --- | --- | --- | --- | --- | --- |
|  |  |  |  |  |  |  |  |
|  |  |  |  |  |  |  |  |
|  |  |  |  |  |  |  |  |

Note: Gender (①male, ②female); Affected by the illness (①yes, ②no)

1. Have you had contact with pigs or other animals? ①No ②Pigs ③Rats ④Dogs ⑤Cats ⑥Other_________________________

Method of contact: ①Slaughtering ②Pets ③Rearing ④Occasional contact ⑤Other___________

5. Do you usually have the opportunity to come into contact with products from the following livestock (pigs, cows, sheep, etc.)? ①Yes ②No

If yes, you can choose multiple options ①Fur ②Offal ③Blood ④Faeces ⑤Raw meat ⑥Cooked meat ⑦Other__________________________________

6. In the 3 months before the illness (if comparing, inquire about the corresponding time with the case), have you had any travel history? ①Yes ②No ③Unclear

(IV) Personal Habits

Personal Habits

1. Have you washed your hands promptly after contact with pets or livestock (pigs, cows, sheep, etc.) products? ①Yes ②No

1.1 Have you used detergent or soap? ①Yes ②No

1.2 Did you have any cuts or abrasions on your hands or arms during the contact? ①Yes ②No

2. Do you wash your hands before and after meals? ①Never ②Occasionally ③Often

3. Do you often share drinking cups and utensils with others? ①Never ②Occasionally ③Often

4. Do you have a habit of drinking untreated water? ①Never ②Occasionally ③Often

5. What are the cooking methods used for several commonly consumed aquatic or marine products?

5.1_____________ ①Steaming ②Stir-frying ③Raw consumption ④Boiling ⑤Other

5.2_____________ ①Steaming ②Stir-frying ③Raw consumption ④Boiling ⑤Other

5.3_____________ ①Steaming ②Stir-frying ③Raw consumption ④Boiling ⑤Other

6.Do you frequently consume alcohol? ①Yes ②No If the answer is yes (estimated based on daily beer consumption)

6.1 Specific frequency ①Frequently (1-2 times/day) ②Occasionally (1-2 times/week) ③Rarely (1-2 times/month) ④Do not drink alcohol

7. How often do you clean your home? ①Clean daily ②1-2 times a week ③Clean when dirty

8. How often do you consume pork at home? ①1-2 times per week ②1-2 times per month ③Very rarely ④Never

9. In the last 3 months, how often did you ventilate your home by opening windows? ①Almost every day ②1-2 times per week ③Very little ventilation.

(V)Basic Health Condition

1. Height: height ℃, Weight: kilograms;

2. Do you suffer from chronic diseases (diagnosed by medical institutions):  ①Yes ②No

① Asthma ②Chronic bronchitis ③Tuberculosis ④Diabetes ⑤ Hypertension

⑥ Heart disease (please specify): ____________________

⑦ Kidney disease (please specify): ____________________

⑧ Chronic liver disease (please specify): _____________________

⑨ Immunodeficiency ⑩ Other chronic diseases (please specify):_____________________

3. Do you suffer from chronic diseases (undiagnosed by medical institutions): ①Yes ②No

① Asthma ② Chronic bronchitis ③ Tuberculosis ④Diabetes ⑤ Hypertension ⑥ Heart disease (please specify): ____________________⑦ Kidney disease (please specify): ____________________

⑧ Chronic liver disease (please specify): ____________________

⑨ Immunodeficiency ⑩Other chronic diseases (please specify): _____________________

4. Do you regularly take the following immunosuppressive drugs: ①Yes ②No

① Prednisone ② Hydrocortisone ③ Dexamethasone ④ Betamethasone ⑤ Other: _____________

(VI)Other Special Findings from the Investigation

____________________________________________________________________

______________________________________________________________________________ ______________________________________________________________________________

(VII)Handling of Epidemic Cases

1. Patient isolation: ①Yes ②No

2. Isolation location: ①Hospital ②At home ③Workplace or school

3. Disinfection of patient's room: ①Yes ②No

4. Disinfection of patient's excreta: ①Yes ②No

5. Disinfectant used: _______________________ and concentration:__________________________

(VIII)Survey Summary

________________________________________________________________________

_______________________________________________________________________________

_______________________________________________________________________________

_______________________________________________________________________________

Investigating Authority:__________________________________________

Investigator: ________ Investigation Time: ____year_____month_____day

Verifier (Data Entry):________ Verification Time: ____year_____month_____day
